# Elucidating the phenotypic landscape of neurodegeneration using multiplexed single‐cell sequencing of defined neuronal co‐cultures *in vitro*

**DOI:** 10.1002/alz.090976

**Published:** 2025-01-03

**Authors:** Mathini Vaikunthan, José McFaline‐Figueroa

**Affiliations:** ^1^ Columbia University, New York, NY USA; ^2^ Columbia University, NY, NY USA

## Abstract

**Background:**

Many putative factors may contribute to the neurodegeneration seen in Alzheimer’s Disease (AD), including the build‐up of toxic amyloid‐beta plaques and the aberrant reactivity of non‐neuronal cell types such as astrocytes and microglia. However, the precise contribution of these factors to normal and disease states of neurons remains poorly defined.

**Method:**

We employed *in vitro* rat neural co‐culture models to determine how changes in cell interactions alter the transcriptional response of neural cell types to agents associated with neurodegeneration. We performed single‐cell RNA‐seq using the 10x Genomics Chromium on neuron‐enriched (Neu) fractions alone or with the addition of an astrocyte‐enriched (Ast) fraction or both astrocyte‐ and microglia‐enriched (Ast + Mic) fractions. We then exposed different cell‐type composition conditions to AD‐related (i.e., Aβ42 aggregates), oxidative/nitrosative (hydrogen peroxide, sodium nitroprusside), and inflammatory/anti‐inflammatory (tumor necrosis factor ɑ, transforming growth factor β, interleukin‐4, lipopolysaccharides, short chain fatty acids) compounds, yielding 20 conditions (including controls). We use multiplexed sci‐RNA‐seq to process all conditions within one experiment. Ongoing work includes gene knockouts of AD relevant genes with CRISPR‐Cas9 Ribonucleoprotein particles before exposure to compounds.

**Result:**

The initial coculture experiment yielded transcriptomes for ∼12k cells. We show that variations in cell‐type composition led to changes in the expression of genes associated with neurodegeneration, including 11 late‐onset AD (LOAD) genes, suggesting that neurodegenerative gene expression changes can occur as a function of altered neural cell interaction alone. Changes in early‐onset AD (EOAD) genes in specific cell‐types were also observed, including increasing *Psen1* expression in neurons upon cell‐type composition changes. Our multiplexed 20‐condition experiment yielded ∼40k transcriptomes. MultiNicheNet–a pipeline for analyzing cell‐cell interactions over multiple conditions–inferred downregulation of LOAD‐associated receptor‐ligand pairs such as *Apoe‐Sorl1* in the context of the “high microglia” coculture model over a number of exposures, recapitulating a transcriptional phenotype seen in AD patients.

**Conclusion:**

By enacting simple perturbations to cell‐type composition and exposures in a neuronal model, we have modeled a subset of the phenotypes displayed in AD patients. This study can help elucidate the order of events of changes observed in AD, including those likely to directly induce neuronal cell death.

